# Celecoxib prevents pressure overload‐induced cardiac hypertrophy and dysfunction by inhibiting inflammation, apoptosis and oxidative stress

**DOI:** 10.1111/jcmm.12709

**Published:** 2015-10-29

**Authors:** Chi Zhang, Fan Wang, Yingxia Zhang, Yimin Kang, Haisheng Wang, Mingming Si, Liping Su, Xue Xin, Feng Xue, Fei Hao, Lechu Yu, Jinzhong Xu, Yanlong Liu, Mingming Xue

**Affiliations:** ^1^ Ruian Center of the Chinese‐American Research Institute for Diabetic ComplicationsWenzhou Medical UniversityWenzhouZhejiangChina; ^2^ School of Pharmaceutical ScienceWenzhou Medical UniversityWenzhouZhejiangChina; ^3^ Beijing Hui‐Long‐Guan HospitalPeking UniversityBeijingChina; ^4^ Inner Mongolia Medical UniversityHohhotInner MongoliaChina; ^5^ The Affiliated Wenling Hospital of Wenzhou Medial UniversityWenlingZhejiangChina

**Keywords:** pressure overload, cardiac hypertrophy, celecoxib, apoptosis, inflammation, oxidative stress

## Abstract

To explore the effects of celecoxib on pressure overload‐induced cardiac hypertrophy (CH), cardiac dysfunction and explore the possible protective mechanisms. We surgically created abdominal aortic constrictions (AAC) in rats to induce CH. Rats with CH symptoms at 4 weeks after surgery were treated with celecoxib [2 mg/100 g body‐weight(BW)] daily for either 2 or 4 weeks. Survival rate, blood pressure and cardiac function were evaluated after celecoxib treatment. Animals were killed, and cardiac tissue was examined for morphological changes, cardiomyocyte apoptosis, fibrosis, inflammation and oxidative stress. Four weeks after AAC, rats had significantly higher systolic, diastolic and mean blood pressure, greater heart weight and enlarged cardiomyocytes, which were associated with cardiac dysfunction. Thus, the CH model was successfully established. Two weeks later, animals had impaired cardiac function and histopathological abnormalities including enlarged cardiomyocytes and cardiac fibrosis, which were exacerbated 2 weeks later. However, these pathological changes were remarkably prevented by the treatment of celecoxib, independent of preventing hypertension. Mechanistic studies revealed that celecoxib‐induced cardiac protection against CH and cardiac dysfunction was due to inhibition of apoptosis *via* the murine double mimute 2/P53 pathway, inhibition of inflammation *via* the AKT/mTOR/NF‐κB pathway and inhibition of oxidative stress *via* increases in nuclear factor E2‐related factor‐2‐mediated gene expression of multiple antioxidants. Celecoxib suppresses pressure overload‐induced CH by reducing apoptosis, inflammation and oxidative stress.

## Introduction

Hypertrophic remodelling characterized by enlarged cardiomyocytes and increase in heart size is an adaptation to stress, especially due to pressure overload. Hypertrophy is the leading cause of various cardiovascular diseases, including hypertension, myocardial infarction, valvular disease and cardiomyopathy 1, 2, 3.

Pressure overload‐induced cardiac apoptosis is the initial pathogenic characteristic of CH and remodelling 4. Mature cardiomyocytes are terminally differentiated; therefore, they lack regenerative capacity 5, 6, 7. Once apoptosis is initiated, necrotic cells are replaced by the extracellular matrix (ECM) which impairs myocardial contractility, increases interstitial fibrosis, promotes CH, and leads to heart failure 8, 9, 10.

Research suggests that cardiac inflammatory mediators expressed in response to pressure overload induces deleterious changes in the cardiac ECM structure and promotes CH 11, 12, 13. In addition, oxidative stress induces mitochondrial‐derived reactive oxygen species (ROS), which activate diverse hypertrophic signalling kinases such as mitogen‐activated protein kinase (MAPK), and transcription factors including NF‐κB, but also increases ECM protein accumulation 14. Therefore, to prevent CH, an ideal therapy would suppress inflammation, oxidative stress as well as apoptosis.

Celecoxib, a cyclooxygenase‐2(COX‐2)‐selective non‐steroidal anti‐inflammatory drug, approved to treat inflammation associated with rheumatoid arthritis and osteoarthritis 15, 16, 17 has been studied *in vitro* and shown to have anti‐inflammatory effects in the vascular endothelium. In addition, strong evidence indicates that celecoxib also played an important role in relieving oxidative stress induced by smoking or ischemia/reperfusion *via* upregulating the expression of multiple antioxidants 18, 19. However, celecoxib's contribution to cessation of apoptosis is controversial. Some reports suggest that celecoxib was considered an apoptosis inducer that prevented tumour formation 20; however, another report confirmed that celecoxib prevented curcumin‐induced apoptosis in a haematopoietic cancer cell model 21.

Research recently confirmed that celecoxib can have therapeutic effects on the heart. Specifically, celecoxib prevented cardiac remodelling in mice with inherited dilated cardiomyopathy 22. Additionally, an *in vitro* study indicated that celecoxib not only was anti‐inflammatory in the vascular endothelium 23 but it also reduced cardiac cell hypertrophy and fibrosis induced by angiotensin and aldosterone 24. However, whether celecoxib can prevent pressure overload‐induced CH and cardiac dysfunction is unclear. Thus, we created a CH rat model using AAC surgery and investigated the effects of celecoxib and we measured the potential protective capacity of celecoxib and its putative association with suppression of inflammation, apoptosis and oxidative stress.

## Materials and methods

### Ethics statement

The protocol was approved by the Institutional Animal Care and Use Committee of the Wenzhou Medical University, Zhejiang, China. All surgery was performed under sodium pentobarbital anaesthesia, and all efforts were made to minimize suffering of the experimental animals.

### Creation of cardiac hypertrophic rat model with AAC surgery

Male Wistar rats [10 weeks‐of‐age, 230 ± 22 g of body weight (BW)], were obtained from the Experimental Animal Center of Beijing University of Medical Science (Beijing, China) and housed in the Experimental Animal Center of Wenzhou Medical University at 22°C with a 12 hrs/12 hrs light/dark cycle, with free access to rodent chow and tap water.

After 2 weeks of acclimation, AAC surgery was performed to induce pressure overload‐induced CH 25. Briefly, the rats were anaesthetized with a 2% sodium pentobarbital solution administered intraperitoneally at a dose of 40 mg/kg BW. A small incision was then made 1‐cm below the xiphoid process and the abdominal aorta was isolated above the renal artery crotch and constricted by a 4‐0 silk suture ligature tied against a 7‐gauge needle. The needle was removed to form a 0.7 mm diameter constriction, which caused 70% arterial stenosis. For the sham operation (SO) group, incisions were made in the chest at the same location as that in AAC rats without aortic constriction. During anaesthesia, the body temperature, respiratory rate and blood circulation of rats were carefully monitored. Four weeks after surgery, we concluded that the CH model was successful as we noted increased heart weight (HW), larger cardiomyocytes, increased, LV mass, great blood pressure and impaired cardiac function.

### Celecoxib treatment

Rats were divided into three treatment groups: sham rats (SO: Group 1); CH control rats (Group 2) and CH/celecoxib‐treated rats (Group 3). Group 3 received celecoxib [2 mg/100 g body‐weight(BW)] daily *via* an intragastic tube for 2 or 4 weeks 26, 27. Group 1 SO rats), and Group 2 CH controls received an equal volume of normal saline. We then measured (BP), other parameters and cardiac function. All animals were then killed at either the 2‐week or 4‐week end‐point under sodium pentobarbital anaesthesia. Heart tissue was collected. The ratios of HW‐to‐BW (HW/BW) ratio and the HW‐to‐tibia length (HW/TL) were recorded at the time of tissue collection.

### Non‐invasive BP

BP readings (systolic pressure, SP; diastolic pressure, DP; and mean pressure, MP) were measured in all animals using tail‐cuff manometry and a BP‐300A non‐invasive BPs monitoring system (Kent Scientific Corporation, Torrington, CT, USA) at each time‐point. Rats were kept warm on a 37°C heating pad to ensure sufficient tail blood flow tail, and then animals were restrained in a plastic tube restrainer where occlusion and volume‐pressure recording cuffs were placed over their tails. Each rat was allowed to adapt to the restrainer for 5 min. prior to BP measurement. Rats were trained for BP measurement over 3 days with 10 acclimation cycles followed by 20 measurement cycles 28.

### Echocardiography

Transthoracic echocardiography (Echo) with a high‐resolution imaging system for small animals (Vevo 770; VisualSonics, Toronto, ONT Canada), equipped with a high‐frequency ultrasound probe (RMV‐707B; VisualSonics) was performed on all rats after they were anaesthetized with 1.2% 2,2,2‐Tribromoethanol (ip). Rat hair was removed from the chest region using a chemical hair remover, and bubble‐free aquasonic clear ultrasound gel (Parker Laboratories, Fairfield, NJ, USA) was applied to the surface of the thorax to optimize cardiac chambers. Parasternal long‐axis and short‐axis views were acquired. Ejection fraction (EF) and fractional shortening (FS) percentages and, LV anterior wall thickness in end‐diastole (LVAWd), LV posterior wall thickness in end‐diastole (LVPWd), LV end‐diastolic diameter (LVIDd) were calculated by Vevo 770 software. The final data represent the average values of 10 cardiac cycles 29.

### Morphological examination of cardiac myocardium

After killed, hearts were removed and washed in cold saline. The entire LV tissue was isolated and fixed with 10% formalin for 2 days at room temperature. After dehydration in ethanol, the tissue blocks were embedded in paraffin and, 3 μm thick sections were made and stained with haematoxylin and eosin for general morphological examination 30. Cardiac fibrosis was examined with 0.1% Sirius‐red F3BA and 0.25% Fast green FCF to assess the collagen accumulation, as previously described 31. Collagen content was quantified using Sirius‐red positive areas and Image Pro software (Media Cybernetics, Silver Spring, MD, USA).

### Terminal deoxynucleotidyl transferase‐mediated dUTP nick end labelling assay

For terminal deoxynucleotidyl transferase‐mediated dUTP nick end labelling (TUNEL) staining, slides were stained with the ApopTag Peroxidase *in situ* Apoptosis Detection Kit (Chemicon, Temecula, CA, USA) 32. Each slide was deparaffinized and rehydrated, then treated with proteinase K (20 mg/l) for 15 min. Endogenous peroxidase was inhibited with 3% hydrogen peroxide for 5 min., and then incubated for 1 hr with the TUNEL reaction mixture containing terminal deoxynucleotidyl transferase (TdT) and digoxigenin‐11‐dUTP. The TdT reaction was carried out in a humidified chamber at 37°C, and 2xSSC was applied, and the mixture was incubated in the dark for 15 min. Counterstaining with 4′,6‐diamidino‐2‐phenylindole was then applied. For the negative control, TdT was omitted from the reaction mixture. Apoptosis was measured by counting TUNEL‐positive cells selected randomly from 10 fields at 40×.

### Western blot

Cardiac tissues were homogenized in lysis buffer (Santa Cruz Biotechnology, Santa Cruz, CA, USA) and supernatants were collected by centrifugation at 12,000 × g at 4°C. Equal amounts of protein from each sample was separated *via* 10% SDS‐PAGE and then transferred to nitrocellulose membranes. After blocking with non‐fat milk for 1 hr at room temperature, the membranes were incubated overnight at 4°C with the following primary antibodies: atrial natriuretic peptide (ANP, 1:1000), brain natriuretic peptide (BNP, 1:1000), β‐myosin heavy chain (β‐MHC, 1:1000), connective tissue growth factor (CTGF, 1:2000), murine double mimute 2 (MDM2, 1:1000), intercellular adhesion molecule‐1 (ICAM‐1, 1:2000), plasminogen activator inhibitor‐1 (PAI‐1, 1:2000), tumour necrosis factor‐α (TNF‐α, 1:1000), nuclear factor E2‐related factor‐2 (NRF‐2, 1:1000), haeme oxygenase‐1 (HO‐1, 1:2000), NAD(P)H:quinone oxidoreductase‐1 (NQO‐1, 1:1000), nuclear factor kappa B (NF‐κB, 1:1000), inhibitor of NF‐κB (IκB, 1:1000) and β‐actin (1:1000), which were purchased from Abcam (Cambridge, MA, USA). Phosphorylated‐P53 (p‐P53, 1:1000), total‐P53 (t‐P53, 1:1000), cleaved‐caspase3 (C‐cas3, 1:500), kelch‐like ECH‐associated protein‐1 (Keap‐1, 1:1000), mammalian target of rapamycin (mTOR, 1:1000), protein kinase B (AKT, 1:1000), phosphorylated‐AKT at ser 473 (1:500), phosphatase and tensin homologue deleted on chromosome ten (PTEN, 1:1000) and phosphorylated‐PTEN (1:500) were purchased from Cell Signaling Technology (Danvers, MA, USA). After three washes in Tris‐buffered saline containing 0.05% Tween 20 (TBST), membranes were incubated with horseradish peroxidase‐conjugated secondary antibodies for 1 hr at room temperature. Antigen–antibody complexes were then visualized using an enhanced chemiluminescence kit (Amersham, Piscataway, NJ, USA), and the intensity of the protein bands was quantified using Quantity one software (Version 4.6.2; Bio‐Rad, Hercules, CA, USA).

### RNA isolation and real‐time quantitative PCR

Total RNA was isolated from heart tissue using TRIzol reagent according to the manufacturer's protocol (Invitrogen, Carlsbad, CA, USA). The total RNA in each sample was quantified with a Nanodrop 2000 (Thermo Scientific, San Jose, CA, USA). RNA samples were reverse transcribed into cDNA using a High‐Capacity cDNA Reverse Transcription Kit (PE Applied Biosystems, Foster City, CA, USA). The following primers were used for RT‐PCR. *NQO‐1*: forward, 5′‐GAGAAGAGCCCTGATTGTACTG‐3′; and reverse, 5′‐ACCTCCCATCCTCTCTTCTT‐3′; *HO‐1*: forward, 5′‐CTCCCT GTGTTTCCTTTCTCTC‐3′; and reverse, 5′‐CTGCTGGTTTCAAAGTTCAG‐3′; β*‐actin*: forward, 5′‐AGGTATCCTGACCCTGAAGTA‐3′; and reverse, 5′‐CACACGCAGCTCATTGTAGA‐3′. RT‐PCR was carried out in triplicate using the SYBR GREEN PCR master mix (Invitrogen) on a Stratagene MX3000p thermocycler (Agilent StrataGene, Santa Clara, CA, USA). The amount of mRNA was calculated by the comparative CT method, which depends on the ratio of the amount of target genes to reference gene β*‐actin*.

### Statistical analysis

Data were collected from all rats (*n* = 8/group) and presented as means ± S.D. One‐way anova was used to assess general differences, followed by a post‐hoc Tukey's test for difference between groups, using Origin 7.5 software for laboratory data analysis and graphing. Statistical significance was considered *P* < 0.05. The Kaplan–Meier method was used for survival analysis.

## Results

### Evaluation of the CH rat model and the effect of celecoxib on arterial BP and LV mass

**Figure 1:**
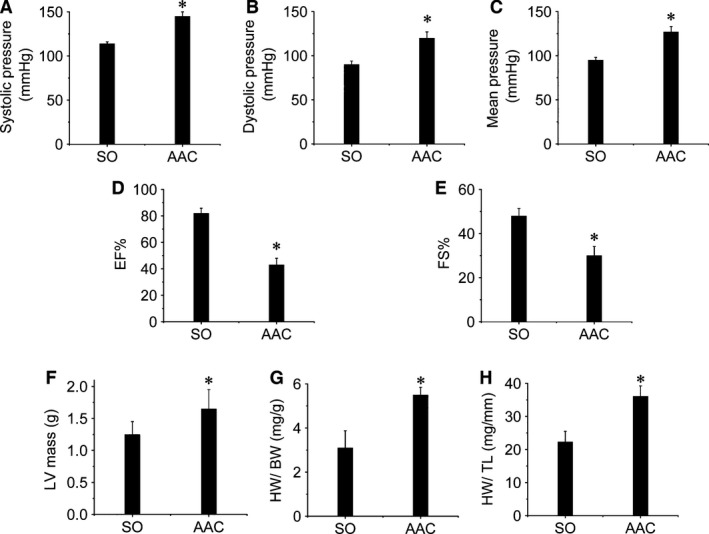
Evaluation of the pressure overload‐induced CH rat model. Four weeks after AAC surgery, CH was evaluated by measuring BP, cardiac function and HW/BW, HW/TL, and LV mass. Occlusion and volume‐pressure recording cuffs were placed over their tails to measure blood pressure, including SP (**A**), DP (**B**), and MP (**C**), by tail‐cuff manometry. Additionally, cardiac function including EF% (**D**) and FS% (**E**) and LV mass (**F**) were investigated by echocardiographic analysis. After the rats were killed, the hearts were removed and weighed followed by calculating the HW/BW (**G**) and HW/TL (**H**). Data are presented as means ± S.D.,* n* = 8 in each group. *P < 0.05 *versus* the SO group. HW: heart weight; BW: body weight; TL: tibia length.

Four weeks after AAC surgery, arterial pressure and cardiac function, and ratios of HW/BW, HW/TL and LV mass of three randomly selected rats from each group were measured to confirm the AAC‐induced CH model. Compared to the SO group, AAC‐treated rats had increased SP (Fig. 1A), DP (Fig. 1B) and MP (Fig. 1C). Meanwhile, cardiac function was impaired as characterized by significant decreases of EF% (Fig. 1D) and FS% (Fig. 1E) in the AAC‐treated rats. In addition, increased HW/BW (Fig. 1F), HW/TL (Fig. 1G) and LV mass (Fig. 1H) was noted in the rats that were AAC‐treated. These data confirmed that the pressure overload‐induced CH was successfully established.

### Celecoxib increased survival rates of rats with CH independent of suppressing hypertension

**Figure 2:**
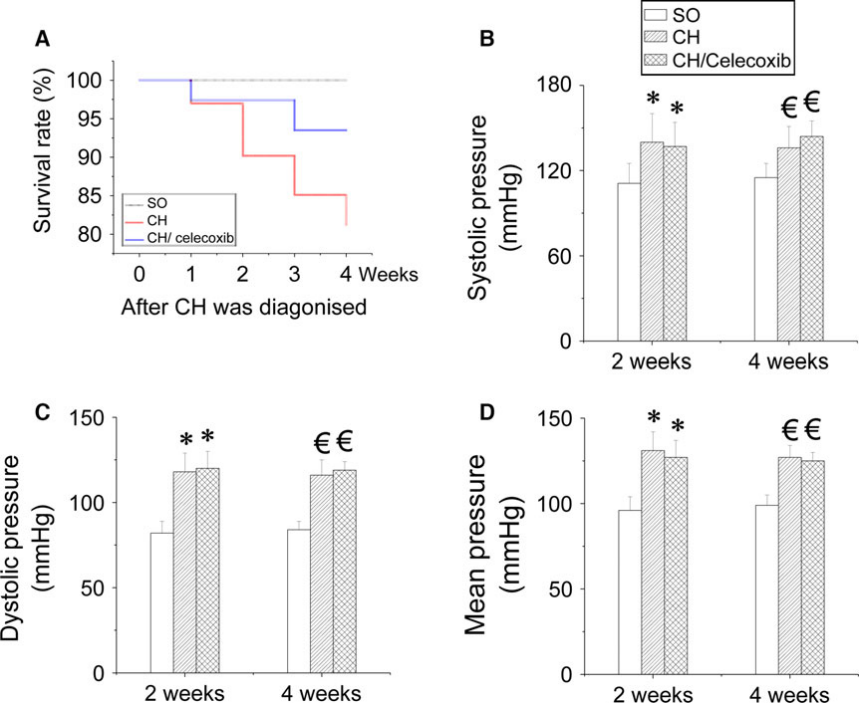
Effect of celecoxib on BP in CH rats. Four weeks after CH was established, three treatment groups were randomized to saline or celecoxib treatment for 2 or 4 weeks. At each time‐point the survival rate of each group was calculated (**A**). Blood pressure including BP, DP and MP were measured using a non‐invasive BP monitoring system (**B**–**D**). Data are presented as means ± S.D.,* n* = 8 in each group. *P < 0.05 *versus* the SO group at 2‐week‐checkpoint; ^€^ *P* < 0.05 *versus* the SO group at 4‐week time‐point. SO: sham‐operated, saline‐treated, Group 1; CH: saline‐treated CH rats, Group 2; CH/celecoxib: celecoxib‐treated CH rats, Group 3.

Cardiac hypertrophy rats were stratified into groups to be treated with or without celecoxib for either 2 or 4 weeks. The survival rates of rats in each group were determined (Fig. 2A). No deaths occurred in the SO group (Group 1) during the experimental period. The survival rate of rats in CH/control group (Group 2) was 90.2% and 81.2% at 2‐ or 4‐week‐time‐point respectively (Fig. 2A). However, CH/celecoxib treatment (Group 3) showed preventive effect on CH‐induced mortality and maintained the survival rate at 97.4% and 93.5% (Fig. 2A). In contrast, celecoxib did not prevent hypertension in CH rats (Fig. 2B–D). Systolic pressure (Fig. 2B), DP (Fig. 2C) and MP (Fig. 2D) strongly increased in both CH and CH/celecoxib groups compared with the SO group. These data show that celecoxib protects against CH, but that this effect is independent of suppression of hypertension.

### Celecoxib prevented AAC‐induced CH cardiac dysfunction

**Figure 3:**
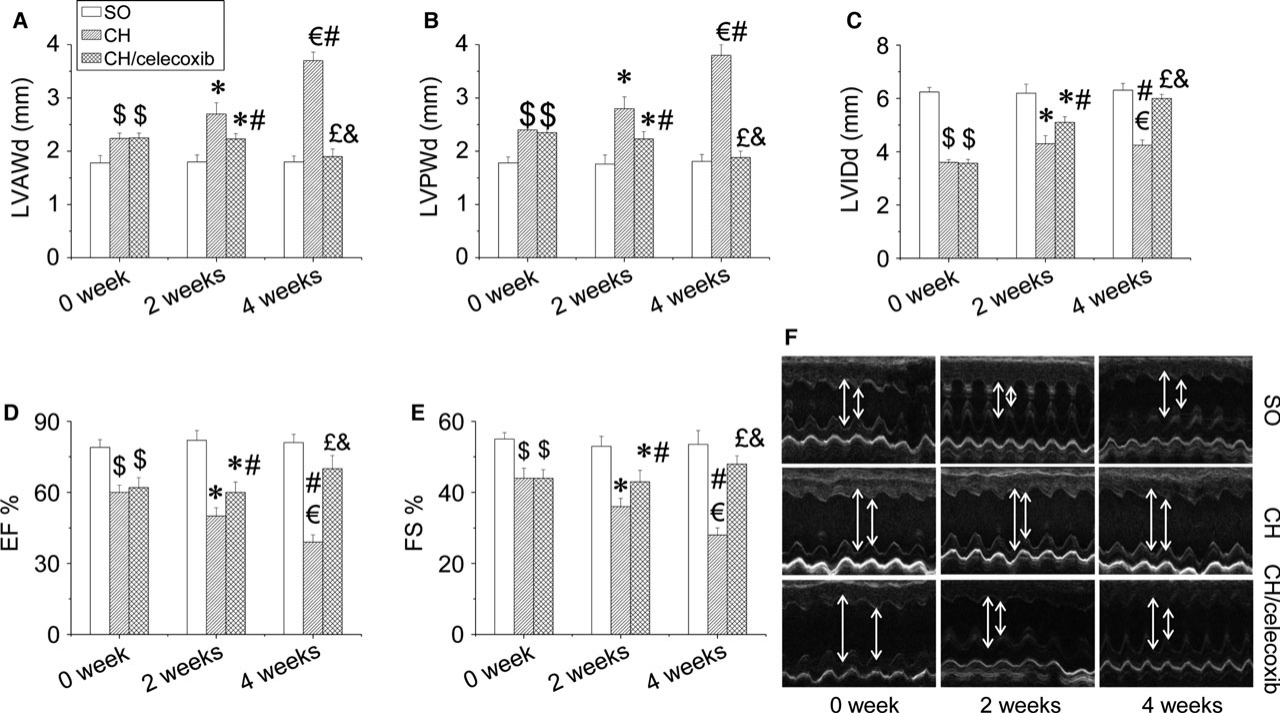
Effect of celecoxib on cardiac dysfunction in CH rats. Cardiac function including LVAWd (**A**), LVPWd (**B**), LVIDd (**C**), EF% (**D**) and FS% (**E**) of rats in each group were evaluated by echocardiographic analysis. Data are presented as means ± S.D.,* n* = 8 in each group. *P < 0.05 *versus* the SO group at 2‐week‐checkpoint; ^€^ *P* < 0.05 *versus* the SO group at 4‐week‐checkpoint; ^#^ *P* < 0.05 *versus* the CH group at 2‐week time‐point; ^£^ *P* < 0.05 *versus* the CH group at 4‐week time‐point; ^&^ *P* < 0.05 *versus* the CH/celecoxib group at 2‐week time‐point. SO: sham‐operated, saline‐treated, Group 1; CH: saline‐treated CH rats, Group 2; CH/celecoxib: celecoxib‐treated CH rats, Group 3.

Next, using the M mode of echocardiography, we studied celecoxib's effect on CH. CH rats after AAC surgery had impaired cardiac function characterized by progressively elevated LVAWd (Fig. 3A and F), LVPWd (Fig. 3B and F), as well as progressively decreased LVIDd (Fig. 3C and F), EF% (Fig. 3D and F) and FS% (Fig. 3E and F). This dysfunction was worsened over time (2 *versus* 4 weeks). Impaired cardiac function in the CH/celecoxib group was comparable to the CH group just after celecoxib treatment. However, the protection on cardiac function of celecoxib was observed after celecoxib treatment at both 2 and 4 weeks (Fig. 3). Statistical analysis indicated that longer treatment offered the better therapeutic effects.

### Celecoxib protected the heart from AAC‐induced hypertrophic remodelling

**Figure 4:**
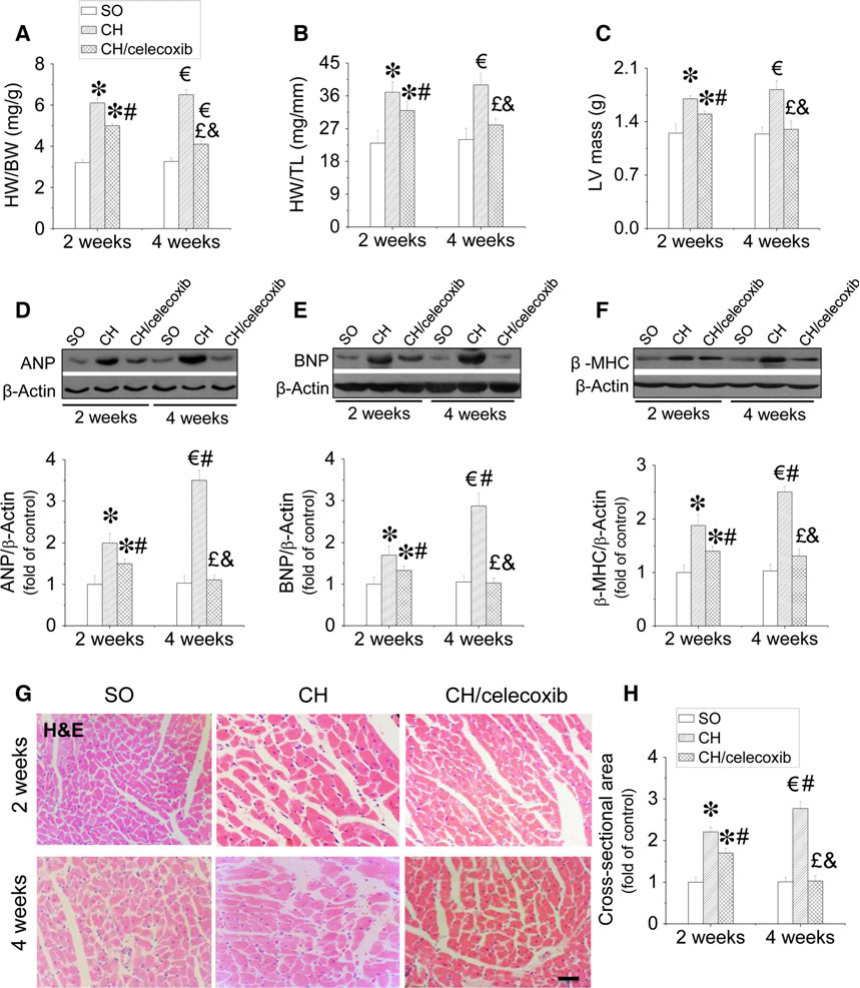
Effect of celecoxib on pressure overload‐induced CH. The CH was evaluated by examining the HW/BW (**A**), the HW/TL (**B**) and the LV mass (**C**), and the expression of cardiac ANP (**D**), BNP (**E**), and β‐MHC (**F**). The cardiomyocyte size was detected by haematoxylin and eosin staining (**G**) and the related calculation of cross‐sectional area (**H**). Data are presented as means ± S.D.,* n* = 8 in each group. *P < 0.05 *versus* the SO group at 2‐week time‐point; ^€^ *P* < 0.05 *versus* the SO group at 4‐week time‐point; ^#^ *P* < 0.05 *versus* the CH group at 2‐week time‐point; ^£^ *P* < 0.05 *versus* the CH group at 4‐week time‐point; ^&^ *P* < 0.05 *versus* the CH/celecoxib group at 2‐week time‐point; bar = 100 μM.

To assess whether the protective effects of celecoxib on cardiac function were attributed to the prevention of CH. We found at 2‐week time‐point, the ratios of HW/BW (Fig. 4A), HW/TL (Fig. 4B), and LV mass (Fig. 4C) were significantly increased, which were further increased at the 4‐week time‐point in the CH saline‐treated rats. In contrast, administration of celecoxib remarkably prevented these increases of hypertrophic parameters (Fig. 4A–C). Additionally, we also examined the expressions of hypertrophic markers at molecular level including ANP (Fig. 4D), BNP (Fig. 4E), and β‐MHC (Fig. 4F). We found cardiac ANP, BNP and β‐MHC expressions strongly increased in CH group at both time‐points, which were remarkably inhibited by celecoxib treatment in a time‐dependent manner (Fig. 4D–F). Morphological analysis with haematoxylin and eosin staining confirmed that the cross‐sectional area of cardiomyocytes significantly increased in the CH group, which was suppressed by celecoxib treatment (Fig. 4G and H). Furthermore, haematoxylin and eosin staining revealed that celecoxib prevented CH‐induced cardiac pathological changes including disorganized array of the myocardial structure, focal cell necrosis and myofibrillar discontinuation (Fig. 4G).

### Celecoxib induced anti‐fibrotic effect in rats with AAC‐induced CH

**Figure 5:**
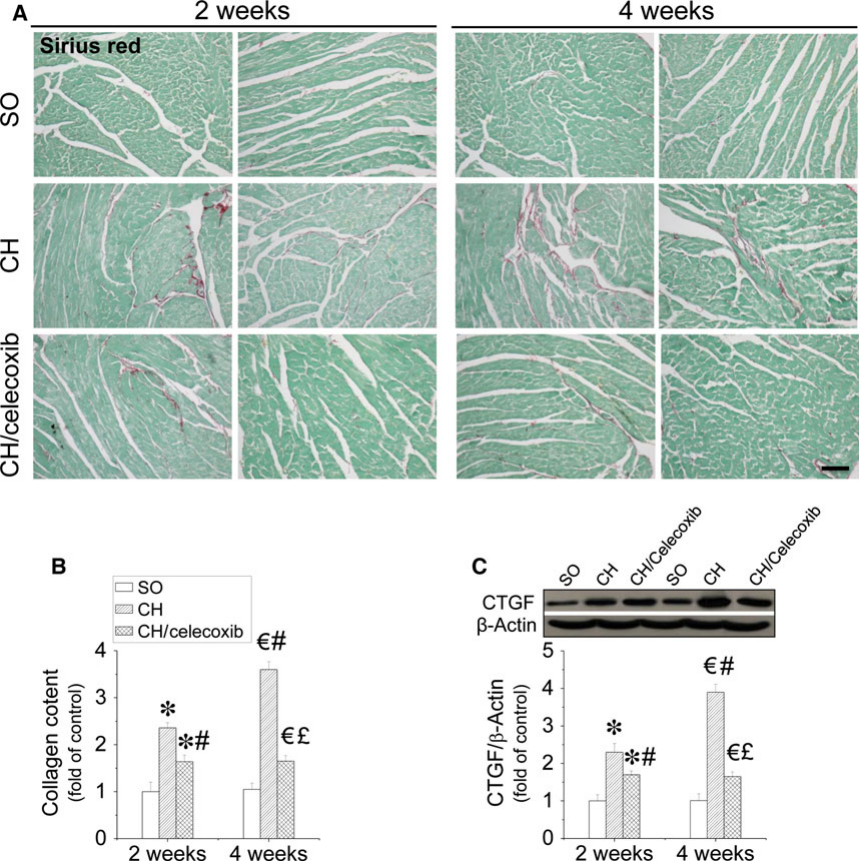
Celecoxib‐induced prevention of CH‐induced cardiac fibrosis *in vivo*. Cardiac sections were subject to Sirius‐red staining with 0.1% Sirius‐red F3BA and 0.25% Fast green FCF for collagen accumulation (**A** and **B**), and also Western blot assay for CTGF protein expression (**C**). Data are presented as means ± S.D.,* n* = 8 in each group. *P < 0.05 *versus* the SO group at 2‐week time‐point; ^€^ *P* < 0.05 *versus* the SO group at 4‐week time‐point; ^#^ *P* < 0.05 *versus* the CH group at 2‐week time‐point; ^£^ *P* < 0.05 *versus* the CH group at 4‐week time‐point; ^&^ *P* < 0.05 *versus* the CH/celecoxib group at 2‐week time‐point; bar = 100 μM.

Because CH is associated with cardiac fibrosis, we examined the fibrotic effect of CH on the heart by Sirius‐red staining for collagen (Fig. 5A and B). Our results showed that CH‐induced significant collagen accumulation, predominantly in the perivascular area, but also in the interstitial tissues, which was increased with the disease process from 2 to 4 weeks. Celecoxib treatment strongly prevented collagen accumulation after 2 weeks treatment, which was more improved at 4‐week time‐point. Additionally, celecoxib induced anti‐fibrotic effects in the heart against CH was also confirmed by suppression of the increased CTGF expression in a time‐dependent manner (Fig. 5C).

### Celecoxib improved cardiomyocyte survival against CH

**Figure 6:**
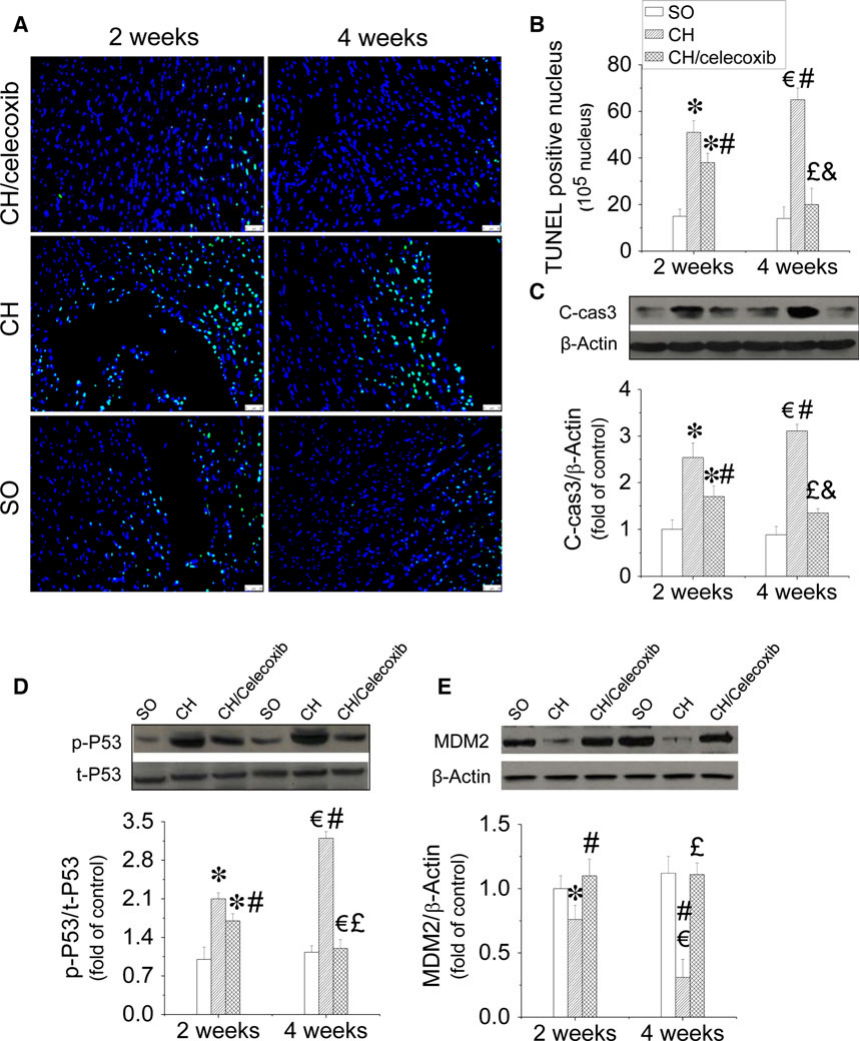
Celecoxib‐induced anti‐apoptotic effects in the hypertrophic heart. After treatment with celecoxib for either 1 or 4 weeks, the rats were killed. Cardiac apoptosis was measured by TUNEL staining using fluorescence microscopy (×40, **A**). The positive nuclei are in green and the cytoplasm is in blue. A semi‐quantitative analysis for apoptotic cells was scored (**B**). The expression cleaved‐caspase 3 was also detected by Western blot assay to further identify cardiac apoptosis (**C**). Both P53 activity (**D**) and MDM2 expression (**E**) were examined by Western blot assay to dissect the anti‐apoptotic mechanism of celecoxib in hypertrophic heart. Data are presented as means ± S.D.,* n* = 8 in each group. *P < 0.05 *versus* the SO group at 2‐week time‐point; ^€^ *P* < 0.05 *versus* the SO group at 4‐week time‐point; ^#^ *P* < 0.05 *versus* the CH group at 2‐week time‐point; ^£^ *P* < 0.05 *versus* the CH group at 4‐week time‐point; ^&^ *P* < 0.05 *versus* the CH/celecoxib group at 2‐week time‐point; bar = 100 μM.

Cardiac apoptosis is an initial pathogenic signal of CH. Thus, we measured cardiac apoptosis using TUNEL staining (Fig. 6A and B) and caspase‐3 cleavage. As shown in Figure 6, increased positive staining for apoptosis was observed in the heart of CH rats at 2‐week time‐point, which was further aggravated at the 4‐week time‐point (Fig. 6A and B). Increased cleaved caspase‐3, an apoptotic marker, was also observed in rats with CH at both time‐points (Fig. 6C). In contrast, administration of celecoxib significantly prevented cardiac apoptosis and improved cell survival after 2 weeks treatment and the anti‐apoptotic effect was much greater after 4 weeks treatment as examined by TUNEL staining (Fig. 6A and B) and caspase‐3 cleavage (Fig. 6C). Since P53 is closely associated with apoptosis, we also investigated the phophorylated‐P53, active form of P53 (Fig. 6D) and the expression of its negative regulator MDM2 (Fig. 6E) in the hearts. We found P53 phosphorylation significantly increased at 2‐week time‐point, which was further enhanced at 4‐week time‐point. Conversely, suppressed MDM2 expression was observed in the hypertrophic heart. In contrast, administration of celecoxib induced time‐dependent inhibition on the activation of cardiac P53, and upregulation of cardiac MDM2 (Fig. 6D and E).

### Effect of celecoxib on inflammation in the AAC‐induced hypertrophic heart

**Figure 7:**
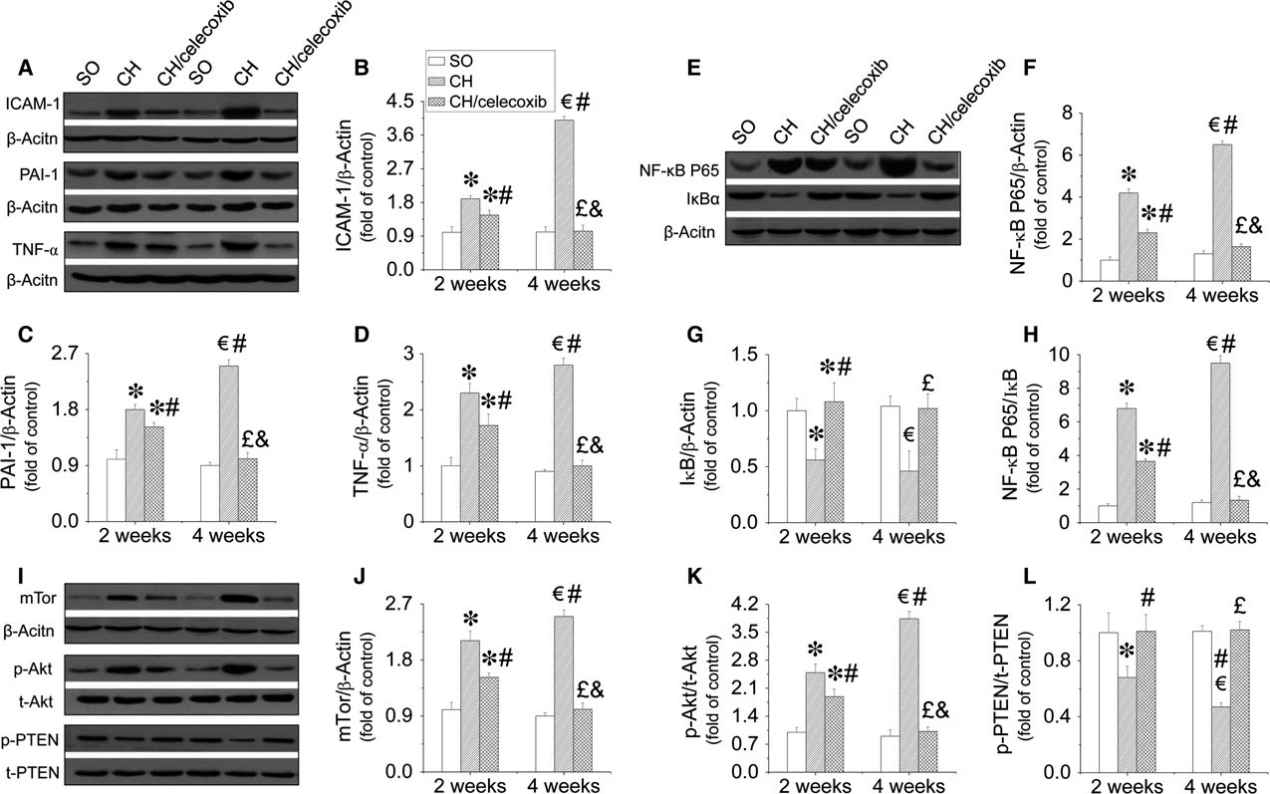
Celecoxib prevented cardiac inflammation in hypertrophic heart *via* inhibition of AKT/mTOR/NF‐κB pathway. Cardiac tissues from all three groups were collected at the indicated times to measure ICAM‐1 (**A** and **B**), PAI‐1 (**A** and **C**), and TNFα (**A** and **D**) by Western‐blot assay. Expressions of NF‐κB (**E** and **F**), IκB (**E** and **G**), and their ratio (**E** and **H**), and mTOR (**I** and **J**) as well as the activity of AKT (**I** and **K**) and PTEN (**I** and **L**) were also examined by Western blot assay to dissect the anti‐inflammatory mechanism of celecoxib in hypertrophic heart. Data are presented as means ± S.D.,* n* = 8 in each group. *P < 0.05 *versus* the SO group at 2‐week time‐point; ^€^ *P* < 0.05 *versus* the SO group at 4‐week time‐point; ^#^ *P* < 0.05 *versus* the CH group at 2‐week time‐point; ^£^ *P* < 0.05 *versus* the CH group at 4‐week time‐point; ^&^ *P* < 0.05 *versus* the CH/celecoxib group at 2‐week time‐point.

Cardiac inflammation was determined by measuring the expressions of the classic inflammatory factors including ICAM‐1 (Fig. 7A and B), PAI‐1 (Fig. 7A and C), and TNF‐α (Fig. 7A and D) in the heart, which were significantly increased in hypertrophic hearts in a time‐dependent manner compared with the SO rats. However, after 2‐weeks of treatment with celecoxib, anti‐inflammatory effects were observed and were characterized by the inhibition of the above mentioned inflammatory factors, which was further enhanced after 4‐weeks of treatment with celecoxib. NF‐κB is well‐known inducer of CH remodelling caused by pressure overload by activation of an inflammatory pathway 33. We confirmed that expression of cardiac NF‐κB p65 gradually increased along with CH development, which was strongly inhibited by administration of celecoxib in a time‐dependent manner (Fig. 7E and F). In contrast, CH suppressed, while celecoxib increased the expression of IκB (Fig. 7E and G), the antagonist of NF‐κB, in the heart as well as the ratio of NF‐κB/IκB (Fig. 7E and H). In addition, upstream activators of NF‐κB‐mediated inflammatory pathway, the expression of mTOR (Fig. 7I and J) and phosphorylation of AKT at Ser 473 (Fig. 7I and K) were also examined. The results showed that mTOR expression and AKT phosphrylation significantly increased with CH process, which were notably inhibited by celecoxib treatment in a time‐dependent manner. Whereas the activation of PTEN (PTEN phosphorylation at Ser 380), the antagonist of AKT activation, was significantly reduced in the CH‐saline treated rats, and restored to with celecoxib treatment (Fig. 7I and L).

### Celecoxib prevented CH‐induced oxidative stress in the heart

**Figure 8:**
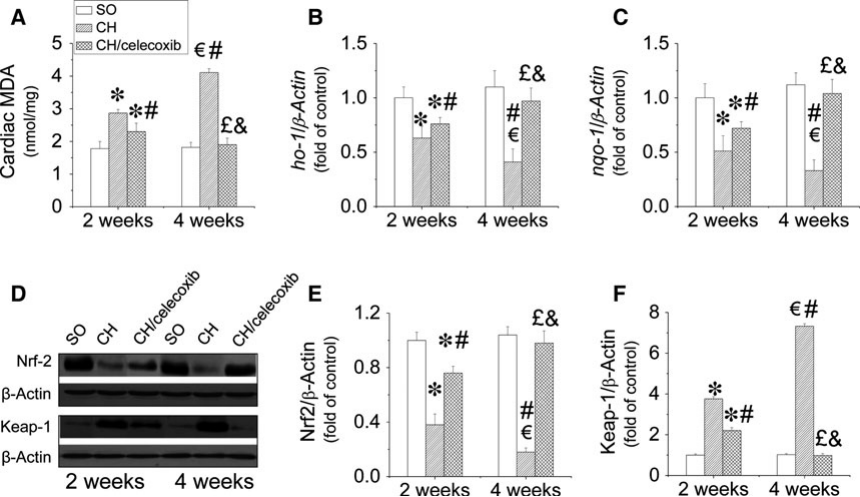
Celecoxib‐induced anti‐oxidative effects in the hypertrophic heart. Cardiac tissues from all three groups were collected at the indicated times to measure cardiac oxidative stress (**A**), and the gene expressions of antioxidants including *HO‐1* (**B**) and *NQO‐1* (**C**) by Real‐time PCR assay. Expressions of NRF2 (**D** and **E**) and its antagonist Keap‐1 (**D** and **F**) were also examined by Western blot assay to dissect the anti‐oxidative mechanisms of celecoxib in the hypertrophic heart. Data are presented as means ± S.D.,* n* = 8 in each group. *P < 0.05 *versus* the SO group at 2‐week time‐point; ^€^ *P* < 0.05 *versus* the SO group at 4‐week time‐point; ^#^ *P* < 0.05 *versus* the CH group at 2‐week time‐point; ^£^ *P* < 0.05 *versus* the CH group at 4‐week time‐point; ^&^ *P* < 0.05 *versus* the CH/celecoxib group at 2‐week time‐point.

Oxidative stress is a key contributor to the development of CH, which was confirmed in our study. Malondialdehyde, marker of oxidative stress, significantly increased in hypertrophic hearts at the 2‐week time‐point, which was further enhanced at the 4‐week time‐point (Fig. 8A). However, this phenomenon was time‐dependently inhibited by celecoxib treatment. Moreover, we found celecoxib inhibited the gene expression decreases encoding for multiple antioxidants including HO‐1 (Fig. 8B), NQO‐1 (Fig. 8C), as well as their transcription factor NRF‐2 (Fig. 8D and E). In contrast, the expression of Keap‐1, negative regulator of NRF2, increased in the hypertrophic heart during the development of the disease, which was significantly inhibited by celecoxib treatment in a time‐dependent manner (Fig. 8D and F).

## Discussion

Cardiac hypertrophy is beneficial to the stressed heart during the early stage, and is characterized by the enlargement of cardiomyocytes size to ensure adequate cardiac function 34. However, these compensatory benefits are temporary, if the stress is not relieved, followed by appearance of cardiac remodelling and cardiac dysfunction 35. The transition from the compensatory to the decompensatory stage is characterized by increases in cardiac fibrosis, apoptosis, inflammation and oxidative stress that eventually lead to heart failure 36, 37, 38.

Cardiac hypertrophy can be induced by various stresses, and pressure overload is one of the most common causes 39, 40, 41. Thus, we established a rat model of pressure overload‐induced CH with AAC surgery, a repeatable technique that offers low mortality 42, 43. During this procedure, constriction percentage and time‐points are key factors for CH development. If aortic constriction is too narrow, the animal may die before cardiac remodelling occurs. Constricting 70–80% of the abdominal aorta for 4 weeks has been established as the ideal time‐point to develop CH in rats 35. Increased blood pressure indicated AAC‐induced pressure overload model was successfully established (Fig. 1A–C). Strong evidence has previously indicated that CH becomes more observable generally 4 weeks after AAC surgery 44, 45, which was confirmed in the present study as characterized by cardiac dysfunction (Fig. 1D and E), increased HW (Fig. 1F and G) and LV mass (Fig. 1H) 4 weeks after AAC surgery.

The pathogenesis of pressure overload‐induced CH is complex and is generally considered as the consequence of hypertension‐induced inflammation, apoptosis and oxidative stress. Therefore, inhibition of the above pathogeneses may significantly prevent CH onset and the subsequent heart failure.

Celecoxib, an anti‐inflammatory agent indicated for osteoarthritis and rheumatoid arthritis therapy, is the first COX inhibitor specific for COX‐2 46. Both pre‐clinical and clinical studies of osteoarthritis and rheumatoid arthritis have demonstrated the efficacy of celecoxib was superior to placebo treatment 47, 48. Previous mechanism studies indicated that the anti‐inflammatory effect induced by celecoxib was attributed to suppression of AKT/mTOR and NF‐κB‐mediated inflammatory pathways 49, 50. In addition, strong evidence also showed that celecoxib induced anti‐oxidative effects in multiple diseases characterized by downregulation of ROS production, and upregulation of antioxidant levels 23, 51. In cancer research, celecoxib was considered an apoptotic inducer which inhibited the tumour formation and the subsequent metastasis 20. However, a recent study presented a controversial conclusion that celecoxib prevented curcumin‐induced apoptosis in a haematopoietic cancer cells 21. Although celecoxib induces diverse functions including anti‐inflammation, anti‐oxidation and anti‐apoptosis according to previously published studies, whether celecoxib can prevent pressure over‐induced CH is not known. Rofecoxib and valdecoxib, also COX‐2 inhibitors, were removed from the market in the early 2000s due to increased cardiovascular risk. Celecoxib remains on the market as being safe for the heart and thus is the single agent available in the US at this time 52. Moreover, randomized clinical trials have demonstrated that renal and cardiovascular safety of celecoxib has also become apparent, as well as its efficacy, tolerability, and safety in the elderly population 48. Growing evidence even indicated that celecoxib induces beneficial effects, rather than deleterious side effects, against cardiovascular stresses including inhibition of cardiac remodelling, cardiac fibrosis and cardiac inflammation 22, 23, 24. Therefore, in this study, we examined whether celecoxib could prevent pressure overload‐induced CH.

Because CH remodelling is the leading cause of cardiac dysfunction, 53, we confirmed that our CH model was successful and assessed cardiac dysfunction. Two weeks after establishment of the CH rat model significant cardiac dysfunction occurred, including increased LAVWd and LAPWd and significantly decreased LVIDd, EF% and FS%, which was further enhanced after 2 more weeks (Fig. 3). Here, we report for the first time that celecoxib prevents CH‐induced cardiac dysfunction in a time‐dependent manner (Fig. 3). Further studies revealed that the beneficial effect of celecoxib on cardiac function was attributed to the improvement of CH. The results showed that the pressure overload‐induced CH was significantly inhibited by celecoxib treatment characterized by decreasing HW/BW, HW/TL, cardiomyocytes size, as well as decreasing multiple hypertrophic markers expressions including ANP, BNP and β‐MHC in a time‐dependent manner (Fig. 4).

Although hypertension is an initial cause of pressure overload‐induced CH 54. The beneficial effect of celecoxib on CH did not extend to amelioration of hypertension in our study. It is therefore likely that other mechanisms exist in celecoxib‐induced prevention of cardiac dysfunction in the rats with CH, which we investigated. One of the key early cardiac response to heart stress, especially hypertension, is the apoptosis which leads to cardiac remodelling and fibrosis due to the filling of ECM in the myocardium 55, 56, 57, 58. Thus, reducing cardiac apoptosis may be beneficial to prevent pressure overload‐induced CH. In our study, we also found gradually increased cardiac apoptosis in the heart of rats in CH group along with CH development (Fig. 6A–C). Consistent with our hypothesis, administration of celecoxib significantly prevented apoptosis in hypertrophic heart in a time‐dependent manner (Fig. 6A–C). Mechanistic studies revealed that anti‐apoptotic effects of celecoxib was attributed to inhibition of P53 activity *via* upregulating the expression of MDM2, P53 antagonist (Fig. 6D and E).

Pro‐inflammatory cytokines are involved in the development of CH because they activate the MAPK pathway, decreasing cardiac contractility, and inducing myocardial interstitial fibrosis 59, 60. As a classic anti‐inflammatory agent, in this study, celecoxib remarkably prevented the expressions of multiple inflammatory factors including ICAM‐1, PAI‐1 and TNF‐α in hypertrophic hearts *via* inhibition of the AKT/‐mTOR/NF‐κB signalling pathway in a time‐dependent manner (Fig. 7). In addition, oxidative stress is known to be involved in the process of cardiac hypertrophic remodelling 61. Strong evidence indicated celecoxib induced preventive effect on oxidative stress 62. Similarly, anti‐oxidative effect of celecoxib in hypertrophic hearts was also observed in our study with the mechanism of not only limiting oxidants productions but also activating the transcriptions of various anti‐oxidative genes attributed to upregulation of NRF2 expression (Fig. 8).

## Conclusions

In summary, CH is the early response of heart to stresses, which if left unchecked, is the leading cause of subsequent cardiac fibrosis and dysfunction. Inflammation and the associated apoptosis and oxidative stress are considered the main pathogeneses of CH. In this study, we attempted to identify the cardiac effect of celecoxib, which is currently used as an anti‐inflammatory drug for a variety of non‐cardiac conditions in clinical practice. Our findings demonstrated that celecoxib significantly prevented pressure overload‐induced CH development and cardiac dysfunction *via* inhibition of apoptosis, inflammation and oxidative stress.

## Conflicts of interest

No potential conflicts of interest relevant to this article were reported.

## Author contribution

C.Z., F.W., Y.Z., Y.K., H.W., M.S., L.S., X.X., F.X., F.H., L.Y. and J.X. substantially contributed to research design, or the acquisition, analysis or interpretation of data; C.Z., Y.L. and M.X. drafted the paper or revising it critically; C.Z., F.W., Y.Z., Y.K., H.W., M.S., L.S., X.X., F.X., F.H., L.Y., J.X. Y.L. and M.X. approved the final version of manuscript for submission.
